# Predictors of cessation of exclusive breastfeeding according to the Cox regression model: survey of mothers of children aged 6-12 months, Thiès, Senegal

**DOI:** 10.11604/pamj.2023.46.12.39603

**Published:** 2023-09-11

**Authors:** Mouhamadou Faly Ba, Oumar Bassoum, Maty Diagne Camara, Adama Faye

**Affiliations:** 1 Institute of Health and Development, Cheikh Anta Diop University, Dakar, Senegal; 2 Department of Preventive Medicine and Public Health, Faculty of Medicine, Pharmacy and Odontology, Cheikh Anta Diop University, Dakar, Senegal

**Keywords:** Exclusive breastfeeding, survival analysis, Senegal

## Abstract

**Introduction:**

even though exclusive breastfeeding (EBF) for up to six months is recognised as essential infant care, it is still insufficiently practiced. The objective of this study was to identify predictors of EBF cessation in Thiès.

**Methods:**

this was a survival analysis of data collected using a cross-sectional procedure. Data collection took place from 2^nd^ December 2019 to 21^st^ January 2020. The study population consisted of mothers of children aged 6 to 12 months residing in Thiès and seen at the reference health centre of the Thiès Health District during infant vaccination sessions. The number of subjects was 400 mothers recruited using a systematic survey, with a sampling interval equal to two. Data were collected through a face-to-face interview. Predictive factors were identified using the Cox regression model. The adjusted hazard ratio (AHR) and its 95% confidence interval (95% CI) are calculated.

**Results:**

the average age of the mothers was 27.08 ± 6.34 years. The proportion of mothers who breastfed their child within one hour of birth was 29.25%. The proportion of those who practiced EBF was 41.50%. The incidence density of EBF cessation was 14 person-months per 100 breastfeeding mothers. The median duration of EBF was 5 months. Lack of advice on EBF during antenatal care (AHR=1.42; 95% CI =1.08-1.85), sources of information other than health professionals (AHR =1.51; 95% CI =1.05-2.19), late initiation of EBF, i.e. breastfeeding beyond 24 hours after birth (AHR =1.53; 95% CI =1.02-2.28) and low level of knowledge about EBF (AHR =1.46; 95% CI =1.11-1.92) were significantly associated with early termination of EBF.

**Conclusion:**

the promotion of EBF for up to six months will necessarily involve the promotion of prenatal consultations during which professionals should raise awareness among future mothers.

## INTRODUCTION

Exclusive breastfeeding (EBF) means that the infant receives only breastmilk. No other liquid or solid foods are given, not even water, except for oral rehydration solutions, or drops/syrups of vitamins, minerals or medicines [1]. The World Health Organization (WHO) recommends exclusive breastfeeding for six months and after that time to introduce the consumption of safe and nutritionally adequate complementary foods, while continuing to breastfeed for up to two years [1]. Both short- and long-term EBF has many benefits for the infant and the mother, including reduced costs of infant care and feeding, and reduced occurrence of several infectious diseases. EBF is also effective in reducing the burden of non-communicable diseases such as diabetes, asthma and cardiovascular disease over the years [2]. Appropriate breastfeeding practice is essential for the growth, survival and development of infants [3].

Globally, about 800,000 neonatal deaths are attributed to late initiation of breastfeeding and discontinuation of the EBF. Globally, 10% of morbidity in children under five was due to non-exclusive breastfeeding. According to The Lancet, suboptimal breastfeeding is responsible for an estimated 1.4 million child deaths and 77% of child deaths are due to non-exclusive breastfeeding in the first six months of life. The highest risk of inappropriate feeding in the first 6 months of life occurs in developing countries where 96% of total child mortality is due to suboptimal breastfeeding [4].

The prevalence of EBF worldwide is below the recommendation where only 36% of children under six months are exclusively breastfed [5]. In 2017, 42% of Senegalese children were exclusively breastfed with a median duration of 2 months [6]. This prevalence was 26% in Spain [7], 21% in Tanzania, 30% in Brazil, 45% in Iran [8]. In Ethiopia, 58% of infants under six months of age are exclusively breastfed. The percentage of exclusively breastfed children decreases sharply with age, from 74% of infants aged 0-1 month to 36% of infants aged 4-5 months. Several studies conducted in various locations around the world have shown that factors such as maternal age, marital status, maternal employment, maternal education, birth order of the newborn, receipt of EBF counselling during antenatal care (ANC) and place of delivery are associated with EBF cessation [5,7,9].

However, data on the determinants of EBF cessation before six months are scarce in Senegal. It is in this context that this study is conducted at the Thiès Health Centre. This study focuses not only on the practice of EBF but also on its duration. The objective was to study the predictive factors of EBF cessation among mothers of children aged 6 to 12 months in the health centre of the Thiès health district.

## METHODS

**Study site:** the study was conducted in Thiès city, located 70 km from the Senegalese capital, Dakar. This commune is made up of 3 arrondissement communes: Thiès North, Thiès East and Thiès West [10]. The population of the commune of Thiès is estimated at 384,192 inhabitants, including 6,761 children aged between 6 and 12 months [10]. The Senegalese health system is organised in a pyramid structure with three levels: central, intermediate and peripheral. The health posts and centres are located at the peripheral level (health district). The city of Thiès belongs to the eponymous health district and houses the reference health centre whose role is to serve as a reference for the other health structures (health post, secondary health centres) located in the geographical area of the Thiès health district [11].

**Study design and sampling:** this was a cross-sectional study with an analytical focus that was conducted from 2^nd^ December 2019 to 21^st^ January 2020. The target population consisted of mothers of children aged 6 to 12 months residing in the commune of Thiès. Mothers of children aged 6 to 12 months seen at the reference health centre of the Thiès Health District during infant vaccination sessions represented the source population. Mothers who were unable to speak and those who refused to participate in the study were not included.

The sample size was calculated according to the following formula:


$$n=\varepsilon^{2}\frac{p\left(1-p\right)}{i^{2}}$$

ɛ=1.96 for a first order risk α=0.05; p=42%. This value corresponds to the percentage of children who benefited from the EBF in 2017 in Senegal [6] and i=0,05. The minimum sample size was 374 children aged 6-12 months. This number was rounded up to 400 to maximize the representativeness of the sample. Systematic sampling was carried out; the procedure was to interview every second mother.

**Data collection:** the collection tool was a questionnaire developed after a literature review [12] and structured in four parts:

***Socio-demographic characteristics:*** mother's age, place of residence, mother's level of education, occupation, marital status, partner's level of education, head of household, number of living children, child's sex, child's age, child's date of birth, child's birth rank, number of deliveries, household size, receipt of a family security grant, membership of a mutual health insurance scheme, average household income.

***Maternal and child health:*** number of antenatal care (ANC), advice on EBF during ANC, interval of last pregnancy with previous one, place of delivery, mode of delivery, postnatal care (PNC), advice on EBF during PNC, weighing of the child, vitamin A supplementation, verification tool and date of receipt of vitamin A, receipt of penta3, verification tool and date of receipt of the third dose of pentavalent vaccine (penta 3), source of information regarding breastfeeding.

***Child's feeding:*** child ever breastfed, time spent with child, time breastfeeding after birth, whether child received liquids or food after first feeding, food or liquid received, whether colostrum was given to child; reason for not giving colostrum; whether the child has been breastfed so far; the age of the child when first given solid/semi-solid/liquid food other than breastmilk and whether the mother was influenced by anyone to use food or liquids before 6 months.

***Mothers' knowledge of the EBF (true/false/don't know):*** this part consisted of 10 questions. Each correct answer (true) was worth 1 point and the other answers (false/don't know) were worth 0 points. The mother was considered to have good knowledge when she scored 8 or more.

The questionnaire was tested with 40 mothers of children aged 6 to 12 months who were seen at the immunization sessions. After this pilot study, the collection tool was reviewed and the final version was consolidated with the participation of the management team. Before the survey began, the interviewer was made aware of the objectives and methodology of the study and trained in the technique for administering the questionnaire. An individual face-to-face interview was conducted and took place during the child immunization sessions. The time required for data collection was 49 days.

**Data analysis:** the data were analyzed using R software version 4.0.5. Descriptive statistics were performed by presenting the variables as mean ± standard deviation (quantitative variables) and frequencies (qualitative variables). A survival analysis was performed. The event was the termination of the EBF. The date of origin was the date of birth. The follow-up time is six months after birth. It is censored when the event did not occur during the first six months of life. The incidence density of EBF cessation was calculated and expressed in person-months. The median duration of the EBF was estimated using the Kaplan-Meier method. Finally, the Cox model was used to identify factors predictive of EBF cessation. Variables with a p-value less than or equal to 0.25 in the bivariate analysis (log-rank test) were included in a global model. Then, the top-down stepwise method was applied and consisted in excluding the variables with the large p-values one by one until a model significantly different from the previous model was obtained. The nested models were compared using the maximum likelihood test. Thus, the models before the last one will be compared using the AIC to take the most parsimonious one; in other words the one with the smallest AIC. This model can be considered the most likely. The final model was composed of the following variables: place of residence, membership of a mutual health insurance scheme, number of ANCs, receipt of advice on EBF during ANCs, sources of information on breastfeeding, time to breastfeed after birth and level of knowledge. The results were presented in the form of an instantaneous risk ratio, also known as the adjusted hazard ratio (AHR), and its confidence interval. The instantaneous risk is the risk of a woman with a given characteristic discontinuing the EBF. To test the proportional hazards assumption in the Cox model, the overall test was conducted as well as a visual inspection of the Schoenfeld residuals [13] versus time curve for non-zero slopes. No evidence of statistically significant deviation from the assumptions of the Cox models was found. The significance level was set at 0.05.

**Informed consent statement:** informed consent was obtained from all subjects involved in the study. The study was authorised by the authorities in the area.

**Funding:** this research received no external funding.

## RESULTS

**Figure 1:**
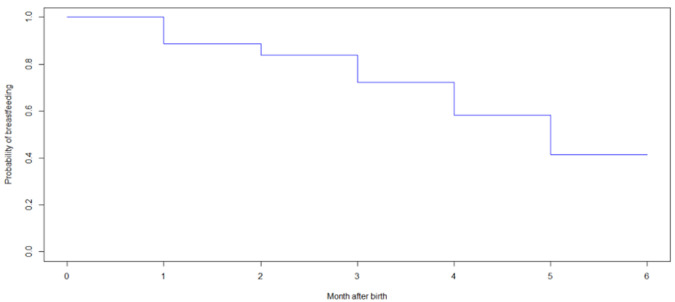
Kaplan-Meier survival curve for exclusive breastfeeding with its median (N=400)

**Descriptive analysis:** in the study, the average age of the mothers was 27.08 years with a standard deviation of 6.34 years. The proportion of mothers surveyed with a higher level of education was 19.00% respectively. The proportion of mothers who put their children to the breast early after birth was 29.25%. The proportion of mothers practicing EBF was 41.50% (Table 1). The incidence density of EBF cessation was 14 person-months per 100 breastfeeding mothers. Figure 1 shows the survival curve for the probability of EBF in the first 6 months of life with the median duration being 5 months. The proportions of EBF in the first and second month were 89% and 84% respectively. At the end, 41.5% were still exclusively breastfed at 6 months.

**Table 1:** distribution of respondents by characteristics

Characteristics	N (%)	Mean ± SD
Mother's age (years)	-	27,08 ± 6,34
Place of residence (Rural)	36 (9,00)	-
**Mother's level of education**		
No	87 (21,75)	-
Primary	117 (29,25)	-
Secondary	120 (30,00)	-
Superior	76 (19,00)	-
**Mother's employment status**		
Employment	168 (42,4)	-
Unemployed	228 (57,6)	-
**Marital status**		
Single	12 (3,00)	-
Divorced	15 (3,75)	-
Married	373 (93,25)	-
**Head of household**		
Me and my husband / Me	41 (10,2)	-
Other	359 (89,8)	-
**Father's level of education**		
No	68 (17,00)	-
Primary	107 (26,75)	-
Secondary	92 (23,00)	-
Superior	133 (33,25)	-
**Number of living children**	-	2,05 ± 1,21
**Gender of the child**		
Female	211 (52,75)	-
Male	189 (47,25)	-
**Birth weight (< 2.5kg)**	39 (9,75)	-
**Household size**	-	11,42 ± 7,73
**Beneficiary of a family security grant**	14 (3,50)	-
**Beneficiary of health coverage**	92 (23,00)	-
**Average household income (≥ 35000Frs)**	344 (86,00)	-
**Number of NPCs (≥ 4)**	265 (66,25)	-
**Receiving advice on the EBF during the ANC**	242 (60,50)	-
**Place of delivery (Public health structure)**	346 (86,50)	-
**Mode of delivery (Lower track)**	334 (83,50)	-
**Receiving advice on the EBF during the PNC**	329 (82,25)	-
**Sources of information on breastfeeding**		
Health professionals	291 (72,80)	-
Vaccination booklet	22 (5,50)	-
Other	52 (13,00)	-
No	35 (8,80)	-
Time spent with the child (Always)	282 (70,50)	-
**Breastfeeding initiation time**		
< 1 hour	117 (29,25)	-
1-24 hours	215 (53,75)	-
24 hours	68 (17,00)	-
Liquids/Food received before 1st feeding	146 (36,50)	-
**Types liquids/foods**		
Ritual water	62 (43,06)	-
Milk other than breast milk	34 (23,61)	-
Other	48 (33,33)	-
Practice of the EBF	166 (41,50)	-
Good level of knowledge about breastfeeding	272 (68,00)	-

**Figure 2:**
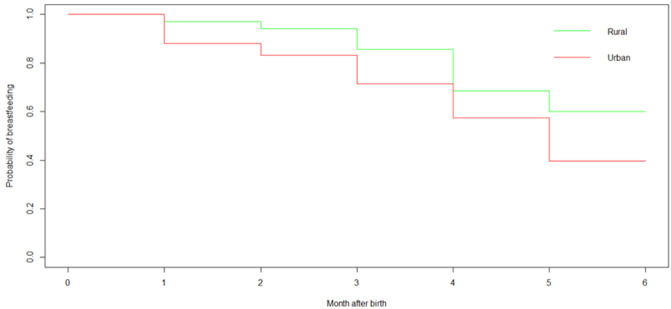
Kaplan-Meier survival curve for exclusive breastfeeding by area of residence (N=400)

**Bivariate analysis:** in bivariate analysis using the log-rank test, factors such as maternal age (p=0.02), area of residence (p=0.03) (Figure 2), number of live children (p=0.03), advice on EBF during ANC (p<0.001), mode of delivery (p=0.006), source of information (p=0.02), time of breastfeeding after birth (p=0.04) and time of birth (p=0.05) were not signifi-cant.0.001), mode of delivery (p=0.006), source of information (p=0.02), breastfeeding time after birth (p=0.04) and level of knowledge about EBF (p=0.001) (Table 2) were significantly associated with EBF cessation.

**Table 2:** analysis of respondents by characteristics

Characteristics	HR brut avec CI à 95%	p-value
**Age of the mother**		
[17-19]	**1,73 [1,13-2,65]**	**0,01**
[20-29]	**1,45 [1,07-1,95]**	**0,02**
[30-45]	**1**	
**Habitat**		
Urban	**1,80 [1,04-2,13]**	**0,03**
Rural	**1**	
**Mother's level of education**		
No	1,01 [0,73-1,35]	0,97
Educated	1	
**Mother's occupation**		
Unemployed	1,11 [0,85-1,45]	0,43
Employment	1	
**Number of living children**		
1-2 children	**1,39 [1,02-1,87]**	**0,03**
≥ 3 children	**1**	
**Gender of the child**		
Female	1,17 [0,85-1,52]	0,22
Male		
**Birth weight**		
≥ 2.5 kg	1,31 [0,81-2,12]	0,27
< 2.5 kg	1	
**Beneficiary of health coverage**		
No	0,76 [0,56-1,02]	0,07
Yes	1	
**Average household income**		
< 35000 FCFA	1,24 [0,86-1,77]	0,24
≥ 35000 FCFA	1	
**Number of ANC**		
< 4	1,30 [0,99-1,69]	0,06
≥ 4	1	
**Receiving advice on the EBF during the ANC**		
No	**1,55 [1,19-2,01]**	**< 0,001**
Yes	**1**	
**Receiving advice on the EBF during the PNC**		
No	1,35 [0,98-1,85]	0,06
Yes	1	
**Mode of delivery**		
Caesarean section	**1,55 [1,13-2,13]**	**0,006**
Lower track	**1**	
**Sources of information on breastfeeding**		
No	1,51 [0,97-2,34]	0,06
Other	1,67 [1,16-2,38]	0,005
Vaccination booklet	1,28 [0,73-2,26]	0,39
Health professional	1	
**Time spent with the child**		
Separate	1,07 [0,81-1,43]	0,60
Always	1	
**Breastfeeding initiation time**		
24 hours	**1,63 [1,10-2,41]**	**0,01**
1- 24 hours	1,29 [0,95-1,76]	0,10
< 1 hour	1	
**Level of knowledge about breastfeeding**		
Low	**1,55 [1,18-2,01]**	**0,001**
Good	**1**	

**Multivariate analysis:** when adjusting for potential confounders by the Cox model, only advice on EBF during ANC (p=0.01), sources of information (p=0.02), breastfeeding time after birth (p=0.03) and level of knowledge (p=0.007) remained significantly associated with EBF cessation (Table 3). Indeed, mothers of children who had not received advice on EBF during ANC were 1.42 times more likely to discontinue EBF than mothers who had received advice on EBF. Next, mothers of children with another source of breastfeeding information were 1.51 times more likely to stop EBF than mothers who received breastfeeding information from health professionals. Then mothers of children who had breastfed their children more than 24 hours after birth were 1.53 times more likely to stop EBF than mothers who had put their children to the breast early after birth. Finally, low knowledge increased the risk of stopping EBF by 1.46 times.

**Table 3:** results of multivariate analysis

Characteristics	Crude HR [CI 95%]	p-value	Adjusted HR [CI 95%]	p-value
**Place of residence**				
Urban	1,80 [1,04-2,13]	0,03	1,43 [0,82-2,50]	0,20
Rural	1		1	
**Beneficiary of a mutual health insurance**				
No	0,76 [0,56-1,02]	0,07	0,75 [0,55-1,02]	0,07
Yes	1		1	
**Number of ANC**				
< 4	1,30 [0,99-1,69]	0,06	1,28 [0,97-1,69]	0,07
≥ 4	1		1	
**Receiving advice on the EBF during the ANC**				
No	1,55 [1,19-2,01]	< 0,001	1,42 [1,08-1,85]	0,01
Yes	1		1	
**Sources of information on breastfeeding**				
No source	1,51 [0,97-2,34]	0,06	1,53 [0,97-2,43]	0,06
Other	1,67 [1,16-2,38]	0,005	1,51 [1,05-2,19]	0,02
Vaccination booklet	1,28 [0,73-2,26]	0,39	1,25 [0,70-2,22]	0,43
Health professional	1		1	
**Breastfeeding initiation time**				
24 hours	1,63 [1,10-2,41]	0,01	1,53 [1,02-2,28]	0,03
1-24 hours	1,29 [0,95-1,76]	0,10	1,22 [0,89-1,69]	0,21
< 1 hour	1		1	
**Level of knowledge about the EBF**				
Low	1,55 [1,18-2,01]	0,001	1,46 [1,11-1,92]	0,007
Good	1		1	

## DISCUSSION

This study showed that discontinuation of the EBF before six months is common practice. Indeed, the overall cumulative probability of exclusive breastfeeding was 41.5%. The median time to discontinue EBF was five months; the predictive factors were low maternal knowledge of breastfeeding, lack of awareness of EBF during ANC, late initiation of breastfeeding, and sources of information other than health professionals.

The low rate of EBF is comparable to the national average found in 2017 (42%) [6], 2018 (46%) [14] and 2019 (41%) [15]. In contrast, the median duration of EBF, i.e. the point at which half of the children stopped being exclusively breastfed, is higher than those estimated in 2017 and 2018; at 2 (DHS 2017) and 2.6 months (DHS 2018) [6,14] respectively. This difference could be explained by the methodology used. DHS is conducted at the community level and targets mothers of children aged 0-23 months. This study is conducted in an institutional setting and its target is mothers of children aged 6 to 12 months.

The link between low levels of knowledge and early cessation of EBF is also evidenced in previous studies conducted in Kenya and Poland [16,17]. Lack of antenatal breastfeeding education is a barrier to EBF. In Mali, a prospective study showed that mothers who had not received counselling on EBF tended to discontinue EBF before the first six months, compared to those who had received it [18]. In addition, mothers seem to trust the information provided by health professionals more than their personal circle. Prenatal education clearly appears to be a source of extrinsic motivation for the initiation and maintenance of EBF. This was also demonstrated in a Canadian study [19].

Although prenatal education on breastfeeding predisposes to the initiation and maintenance of the EBF, it is nevertheless not sufficiently practiced. Indeed, only 60.5% of mothers received advice during ANC. Moreover, 72% have health professionals as their source of information. However, the WHO recognizes the important role that health workers should play in promoting breastfeeding. It has therefore launched the Baby Friendly Hospital Initiative to encourage maternity hospitals to adopt the Ten Steps to Successful Breastfeeding [20].

A Cochrane review has also shown that when breastfeeding support is offered to women, the duration and exclusivity of breastfeeding is increased. According to this review, this support is most effective when offered by health staff during ANC or PNC [21]. This study found that early initiation of breastfeeding was found to be a factor in maintaining EBF up to six months [21]. A prospective Taiwanese study of 300 women indicated that initiation of breastfeeding within 4 hours of birth was a factor associated with discontinuation of the EBF compared to initial breastfeeding within 1 hour of birth [22].

A systematic review by the Cochrane Collaboration has shown that immediate (within 10 minutes of birth) or early (10 minutes and 24 hours after delivery) skin-to-skin contact promotes continued breastfeeding at one to four months after birth [23]. Thus, skin-to-skin contact should be promoted as a crucial time to initiate breastfeeding within one hour of birth. In view of these results, three actions should be carried out: raising awareness among mothers, families and the community about the importance of EBF up to six months. ANCs are an opportunity to do this. In addition, the involvement of the Bajenu Gox would be necessary to carry out home visits to pregnant women. Training of health care providers on good infant feeding practices and interpersonal communication skills. To this end, training manuals are developed to facilitate the learning of health workers [24,25]. Promotion of early initiation of breastfeeding. This requires support from health workers to the mother immediately after delivery. A major opportunity would be skin-to-skin contact [25].

This study has some limitations. Information on breastfeeding practices is based on mothers' self-reports. Therefore, this method of collection could introduce a recall bias. However, the inclusion of mothers of children aged 6 to 12 months was chosen to reduce the risk of this bias. In addition, the study is conducted in an institutional setting. Therefore, it could result in an under-representation of mothers who do not use child immunization services. However, there are strengths to this study that are worth highlighting. Firstly, the use of survival analysis is very relevant. It allowed the identification of groups at risk of early discontinuation of EBF. To our knowledge, this is the first study of its kind in Senegal. This study thus paves the way for future research on the time to discontinuation of EBF.

## CONCLUSION

This study showed that the factors predicting the cessation of EBF before six months are modifiable through three axes. Firstly, it will be necessary to i) raise awareness among mothers of children during ANC, ii) raise awareness among the entourage of mothers of children by means of social mobilization and iii) promote skin-to-skin contact which is an appropriate time to practice immediate breastfeeding. Prospective studies should be conducted on large samples to identify other factors that predict cessation of EBF.

### What is known about this topic

- *Exclusive breastfeeding (EBF) for the first six months of a child's life is crucial for their health and development;*
- *Despite the known benefits of EBF, the prevalence of EBF is low in many parts of the world;*
- *Previous studies have identified several factors associated with early termination of EBF, including maternal education, employment, and socio-economic status*.

### What this study adds

- *This study provides new evidence on the predictors of EBF cessation in Thiès, Senegal, including lack of advice on EBF during antenatal care, sources of information other than health professionals, late initiation of EBF, and low levels of knowledge about EBF;*
- *The study highlights the importance of prenatal consultations in promoting EBF and raising awareness among future mothers;*
- *This study contributes to the growing body of literature on EBF and can inform the development of targeted interventions to promote and support EBF in Thiès, Senegal*.
